# How cyclophosphamide at environmentally relevant concentration influences *Daphnia magna* life history and its proteome

**DOI:** 10.1371/journal.pone.0195366

**Published:** 2018-04-05

**Authors:** Małgorzata Grzesiuk, Damian Mielecki, Tomasz Pilżys, Damian Garbicz, Michał Marcinkowski, Elżbieta Grzesiuk

**Affiliations:** 1 Department of Hydrobiology, Faculty of Biology, University of Warsaw at Biological and Chemical Research Centre, Warsaw, Poland; 2 Institute of Biochemistry and Biophysics Polish Academy of Sciences, Warsaw, Poland; VIT University, INDIA

## Abstract

The waste of commonly used medicines is known to contaminate freshwater ecosystems. Pharmaceuticals can be toxic, mutagenic, or modifying to freshwater organisms even at low concentrations if consider their permanent presence in the environment. Chemotherapeutics used to treat cancer, and in particular alkylating agents, contribute significantly to this form of pollution, the latter introducing cytotoxic and/or mutagenic lesions to the DNA and RNA of organisms which can be disruptive to their cells. The aim of the present study was to investigate the influence of the alkylating anticancer agent cyclophosphamide (CP) on *Daphnia magna* clones. We evaluated the life history parameters and protein profiles of this crustacean following exposure to environmentally relevant CP concentration of 10 ng L^-1^. Even at this low concentration, the alkylating agent caused modification of the life history parameters and proteome profile of the *Daphnia*. These changes were clone-specific and involved growth rate, age at first reproduction, neonate number, and proteins related to cell cycle and redox state regulation. The disturbance caused by pharmaceuticals contaminating freshwater ecosystem is probably weaker and unlikely to be cytotoxic in character due to the high dilution of these substances in the water. However, our results indicate that prolonged exposure of organisms to these toxins may lead to modifications on the organismal and molecular levels with unpredictable significance for the entire ecosystem.

## Introduction

Commonly used pharmaceuticals have been found to be significant pollutants of aquatic ecosystems [1–3]. These substances, including anti-cancer agents, have been detected in the surface waters of many countries due to an increase in the consumption of medicines over the past decade [4–6]. In 2011, the UK Office of National Statistics predicted that the use of pharmaceuticals in Great Britain would at least double by 2050 (UK Office of National Statistics 2011 https://www.ons.gov.uk). This phenomenon is occurring primarily due to the aging of society as well as the increase in frequency of so-called diseases of affluence, such as type 2 diabetes, heart disease, metabolic syndrome, depression, obesity and cancer. Pharmaceuticals enter sewage treatment plants mainly following their consumption and subsequent excretion by people, or directly by flushing the overdue medicines themselves down toilets [7–9].

Alkylating agents, including those used as chemotherapeutics, contribute significantly to the contamination of freshwater ecosystems [10]. This group of chemicals represents the oldest class of anticancer agents to have been developed and is still used despite the introduction of targeted therapies. Alkylating agents modifying DNA, RNA and proteins by alkylation, create byproducts which, if not removed, can be toxic or mutagenic to the cell. At high concentrations they can lead to cell death or, in higher organisms, various kinds of cancer. Cyclophosphamide (CP) is an oxazaphosphorine-type chemical with its main effect on cells caused by its metabolite, phosphoramide mustard formed only in cells with high levels of aldehyde dehydrogenase. Phosphoramide mustard creates DNA crosslinks both between and within DNA strands at guanine N-7 positions, known as interstrand and intrastrand crosslinkers, respectively. This damage is irreversible and leads to cell apoptosis [11]. CP can be used in chemotherapy to treat blood cancer, solid tumours, and sarcoma by targeting the affected cells.

The impact of pharmaceuticals on organisms is often tested in laboratories at higher concentrations (i.e. miligrams per litre) than are found in the environment (i.e. nanograms per litre) [12, 13]. Studies monitoring the concentrations of pharmaceuticals in surface waters have shown them to be low (ng L^-1^); these substances are, however, being released into the water on a constant basis [14]. The scarcity of research into the effects of the low- concentrations of pharmaceuticals found in the environment on so-called non-target organisms, which was highlighted by Fent et al.2006 [15], remained a problem for about a decade. Recently concentrations of pharmaceuticals as present in freshwater ecosystems have come into focus. Medicine have been shown to significantly affect the functioning of aquatic biofilms [16]. Environmental concentrations of pharmaceuticals can also significantly affect primary producers (algae) crossing two trophic planktonic levels, affecting *Daphnia* [17].

*D*. *magna*, a keystone species inhabiting lakes and ponds in Europe, is a model organism which has been frequently used for research on the molecular mechanisms of phenotypic plasticity, adaptation, and microevolution [18, 19]. Its ecology and response to stressors are well-understood by researchers [15–20]. Aim of present study was to examine if the exposure of *D*. *magna* to low concentrations of CP results in modification of its life history parameters and changes in its proteome profiles.

## Materials and methods

### Pharmaceutical materials

Cyclophosphamide monohydrate (CP) was provided by SIGMA (C7397-1G). We used a stock solution containing 1 mg mL^-1^ of CP in de-ionized water. *Daphnia* were exposed to the CP at the concentration normally detected in freshwater ecosystems [4], namely 10 ng L^-1^.

### Daphnia magna clones

We used three *D*. *magna* clones in the experiment, referred to as D, N and S, taken from three separate reservoirs in the same region in order to obtain different genotypes while keeping environmental conditions relatively constant. The clones came from three ponds in the suburbs of Ceske Budejovice, Czech Republic in May 2016: D was obtained from Domin (49.00N, 14.43E), N was from Novy Vrebensky Rybnik (49.00N, 14.44E) and S was from Stary Vrebensky Rybnik (49.01N, 14.43E). There was no need for permission for the collection of animals from specified lakes. To avoid any maternal effect, the clones were cultured for at least 3 generations in the laboratory prior to the experiment. Thus, neonates from the second clutch were used for further breeding and in experiment. The *Daphnia* clones were cultured under controlled, constant conditions: each individual was kept in 100 mL of lake water at a temperature of 20°C, with dim light-imitating lighting in epilimnion. The lake water, which originated from Szczęśliwice (Warsaw, Poland), was aerated for at least 4 weeks and filtered through a 0.2 μm capsule filter prior to use. The *Daphnia* were fed daily with green algae, *Acutodesmus obliquus*, at the non-limiting growth concentration of 1 mg C_org_ L^-1^ [11]. During the experiment, *Daphnia* clones were either cultured without or in the presence of CP (10 ng L^-1^) (control vs experimental conditions). To avoid the problem of CP decay, the experimental medium was changed every second day with a fresh dose of CP. Individuals were cultured for up to 11 days until first reproduction. All clones were tested at the same time.

### Daphnia magna life history parameters

**Fig 1:**
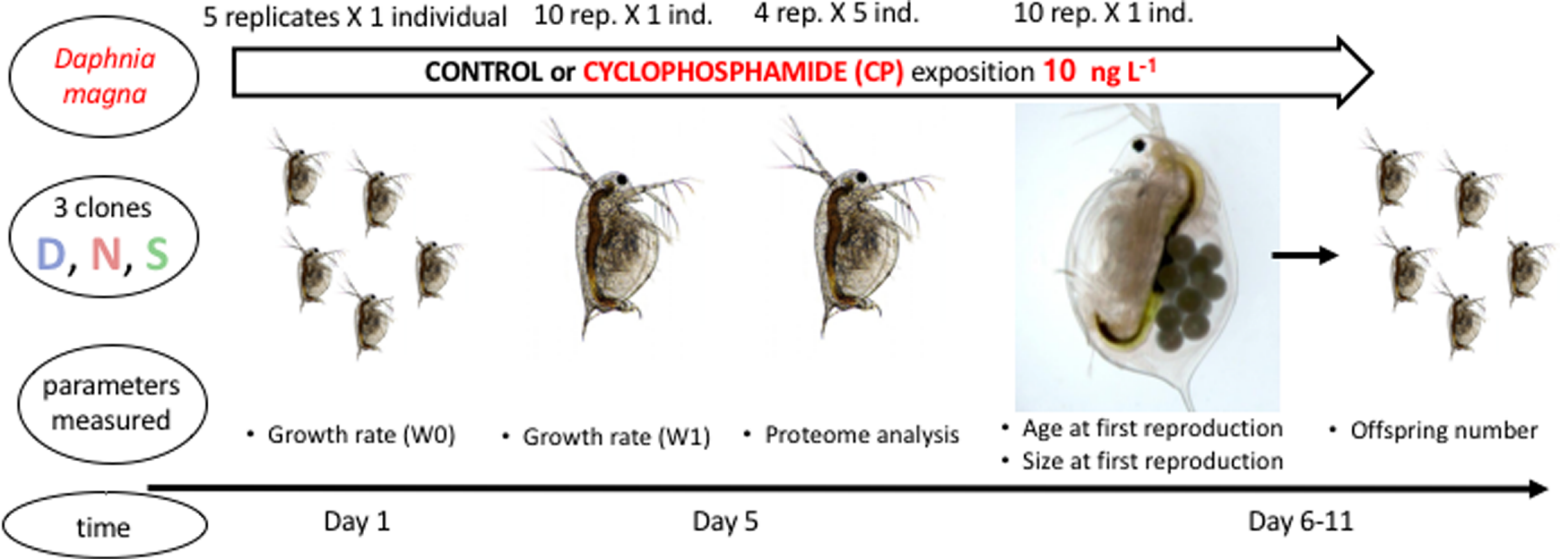
The experimental design (description in the text).

The experimental design is depicted in Fig 1.

Juvenile somatic growth rates were obtained from 5-day old *Daphnia* without eggs. At the beginning of the experiment, five aluminium weighing bottles, each containing three <24-hour old individuals from each clone were dried (24 h; 60°C) and weighed after 24 h. In addition, 10 5 day-old animals from each clone were weighed after 24 h of drying (one individual per weighing bottle). The *Daphnia* were weighed using an Orion Cohn C-35 Microbalance Thermo Scientific (exact to within 0.1 μg). Juvenile somatic growth rates (*g*) were measured via the increase in dry mass of the *Daphnia* between the beginning of the experiment (Wt_0_) and day 5 (Wt_1_), using the equation [20]:

$$g=\frac{\ln W_{t1}-\ln W_{t0}}{t_{1}-t_{0}}$$

Age at first reproduction was noted and number of offspring was counted every 24 h. When they reached the age of first reproduction, the *Daphnia* were photographed and measured using the NIS Nikon System.

### Mass spectrometry analyses of protein profiles

Analyses were performed on juvenile (5-day old) animals without eggs from all three of the *D*. *magna* clones. First, in order to clean their guts, both experimental groups, control and CP-treated, were placed in a medium without food. Each sample was then prepared in quadruplet (except for the treated D clone, which was prepared in triplicate due to a shortage of individuals), each containing 5 *Daphnia*.

The *Daphnia* were then dried on a filter, homogenized and resuspended in a RIPA buffer in the presence of a protease inhibitor cocktail. Cellular debris was spun at 30,130 x g and the resulting supernatant was analyzed for protein content using the Bradford assay [21]. Samples were diluted with SDS-PAGE loading buffer to achieve a final protein concentration of 2 μg μL^-1^, and 10 μL was loaded on the Mini-PROTEAN TGX 4–15% gradient gel (Bio-Rad). Protein bands were cut out of the gel and used for mass spectrometry analysis.

Venn diagrams were generated using webtool (http://bioinformatics.psb.ugent.be/webtools/Venn) and prepared with Gimp 2.8.14 software.

The functions of the proteins were retrieved from the UniProt database [22].

### Statistical analysis

Data were analysed using an ANOVA model. All analyses were carried out using the software programs Statistica 8 and Origin.

## Results

### Growth rate

**Fig 2:**
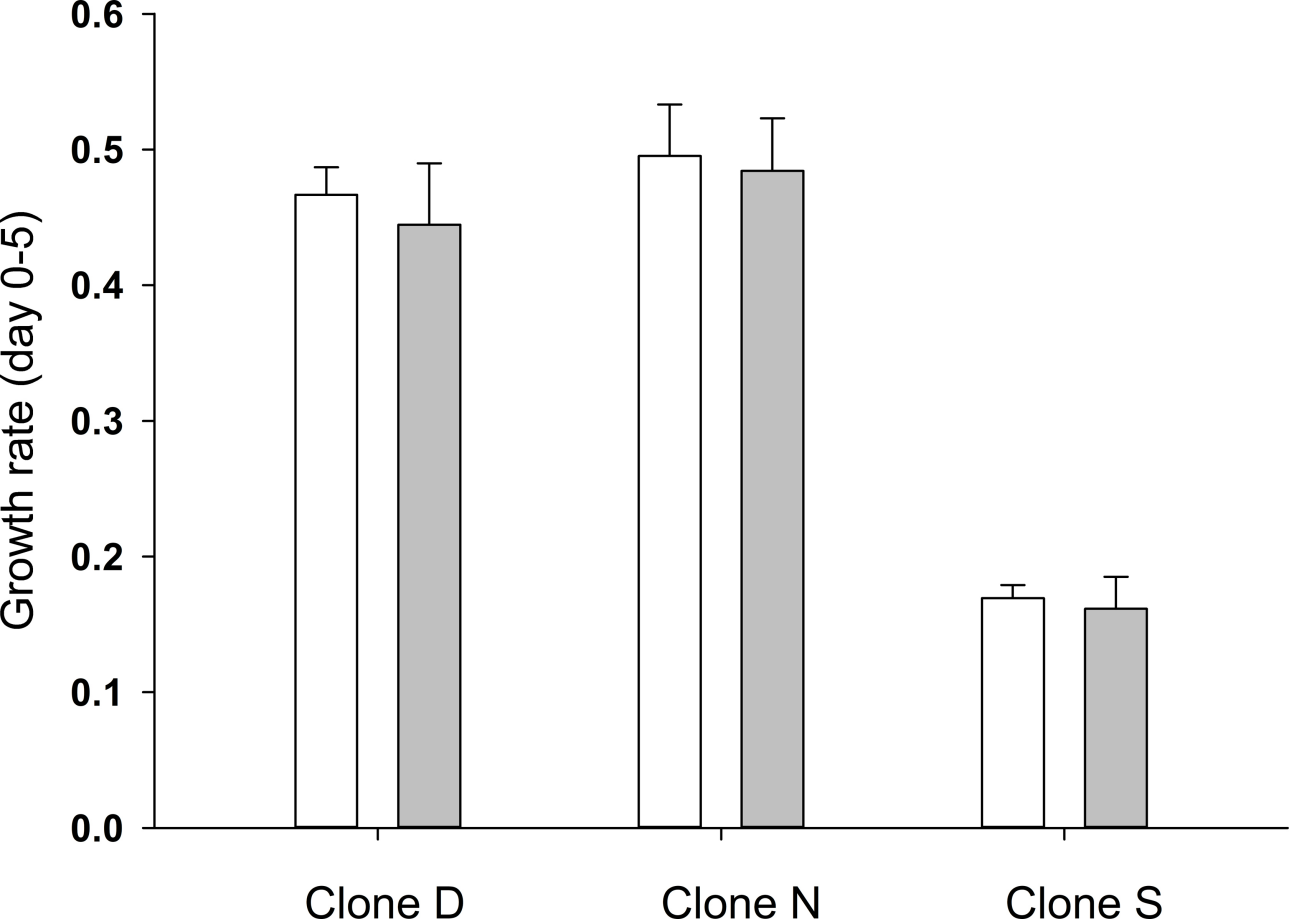
The influence of cyclophosphamide on the growth of *D*. *magna*. (a) Average ± SD growth rate of *Daphnia* clones D, N and e S on Day 5, cultured with (grey bars) or without (white bars) cyclophosphamide.

Compared to the untreated controls, the growth rate of the *D*. *magna* individuals exposed to CP was lower in all three clones, by approx. 5% (clone D), 2.5% (clone N) and 5% (clone S) (Fig 2), although none of these differences were statistically significant (One way ANOVA clone D: F_(1, 17)_ = 1.81, p = 0.196; clone N: F_(1, 17)_ = 0.386, p = 0.542; clone S: F_(1, 15)_ = 0.671, p = 0.425).

### Age at first reproduction

**Fig 3:**
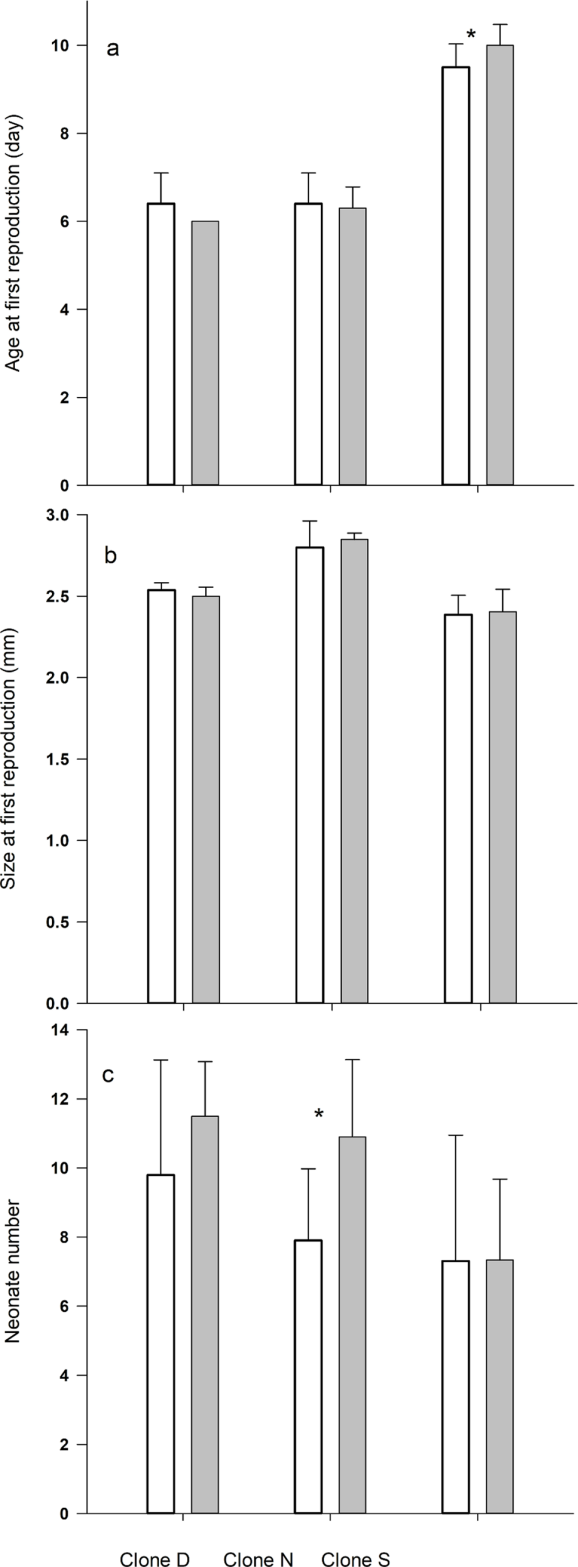
The influence of cyclophosphamide on the reproduction of *D*. *magna*. Average (± SD) (a) age, (b) size at first reproduction and (c) first neonate number of *Daphnia* (Clones D, N and S) cultured with (grey bars) or without (white bars) cyclophosphamide. Stars indicate significant differences between the control and experimental groups.

CP had an effect on the age at first reproduction of clone S (Fig 3A), with the individuals maturing later (10 days old) when cultured with CP compared to those *Daphnia* which were not exposed to the pharmaceutical (9.5 days old) (One way ANOVA: F_(1, 18)_ = 5, p = 0.038). The age at the first reproduction was not affected by the presence of the alkylating agent in the other two clones (6 vs 6.4 days experimental vs control old for clone D and 6.4 vs 6.3 days old for clone N).

### Size at first reproduction

The addition of the pharmaceutical had no significant effect on size at the first reproduction on any of the *D*. *magna* clones tested (Fig 3B) (Clone D: 2.53 mm vs 2.50 mm control vs experimental; Clone N: 2.79 vs 2.84 mm; Clone S: 2.40 and 2.38 mm).

### Number of neonates at first reproduction

*D*. *magna* from clones N, D and S, reacted differently to the CP contamination. The number of first neonates for the N clone was significantly higher (3 more offspring on average, or 38%) when the animals had been treated with CP (One way ANOVA: F_(1, 18)_ = 9.66, p = 0.006) (Fig 3C). Although D clone animals treated with CP had an average of 1.7 (17%) more neonates than those in the non-treated control, this difference was not statistically significant (One way ANOVA: F_(1, 18)_ = 2.13, p = 0.162). S clone animals had an average of 7.3 neonates in the first clutch under both experimental and control conditions (Fig 3C).

### Protein differential analysis

**Fig 4:**
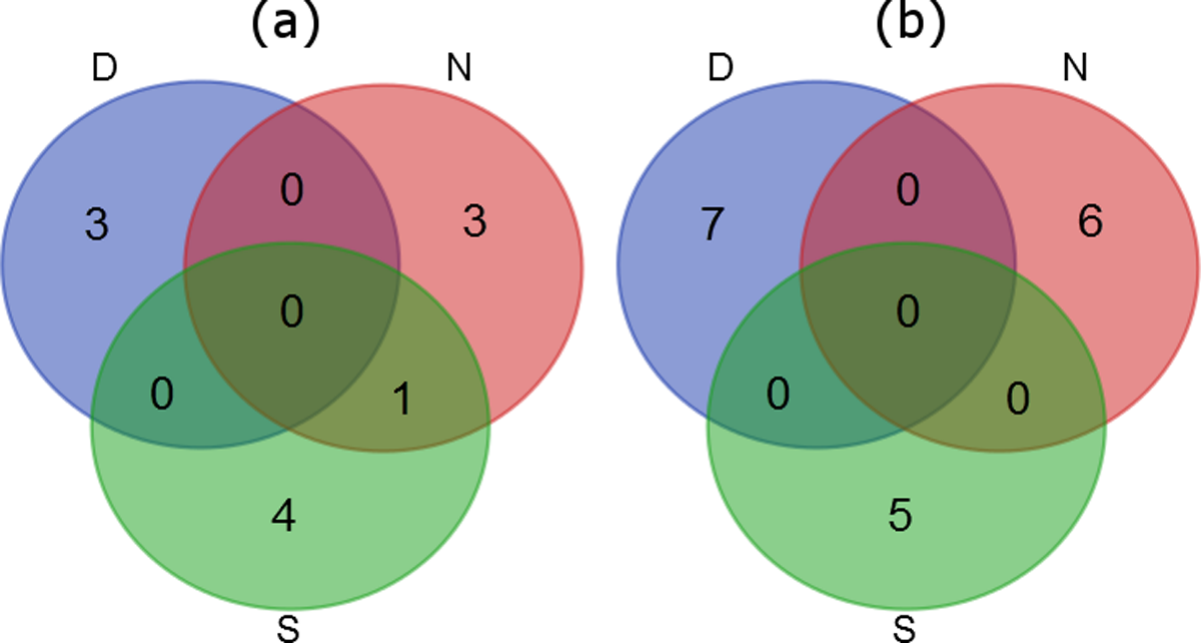
Common proteins with changed levels in *Daphnia* animals treated with cyclophosphamide. A Venn diagram showing common proteins, either (a) up- or (b) down regulated, in *Daphnia*, which changed significantly (q value < 0.1) in the clone D, N and S individuals which had been treated with cyclophosphamide.

In order to identify proteins playing a role in the response of *Daphnia* to genotoxic pollutants, we used mass spectrometry. The analysis identified 1192, 1239 and 1230 proteins from the D, N and S clones, respectively (S1, S2, and S3 Files), of which 10, 12, and 10 proteins were significantly changed (q value < 0.1) or appeared only in the untreated organisms of all clone groups (Fig 4, Tables 1–3). In each clone, only one to three proteins were found to be up- or down-regulated in the presence of the pharmaceutical. With one exception, there were no proteins common to the three treatments (Fig 4).

**Table 1:** Proteins with changed expressions in the D clone individuals which had been treated with CP, compared to control individuals, with q-value < 0.1 (ratio = control (C)/CP treated animals).

no	protein	qvalue	ratio	description
1	KZS16579.1	NA	only in C	Uncharacterized protein APZ42_017126 [Daphnia magna]
2	KZS08626.1	NA	only in C	NADH dehydrogenase [ubiquinone] 1 alpha subcomplex subunit 2 [Daphnia magna]
3	JAN29398.1 KZS21037.1	NA	only in C	Vitellogenin-1 precursor-like protein [Daphnia magna] Uncharacterized protein APZ42_012128 [Daphnia magna]
4	JAN86019.1 JAN30129.1 KZS21347.1 JAN49269.1 JAN31626.1 JAN31627.1 JAN44428.1 (5 more)	NA	only in C	hypothetical protein [Daphnia magna]
5	JAN41153.1 KZS11037.1	NA	only in C	putative Glutathione S-transferase theta-1 [Daphnia magna]
6	JAN56176.1 JAN38323.1 JAN35680.1 JAN35679.1	NA	only in C	Ectonucleotide pyrophosphatase/phosphodiesterase family member, partial [Daphnia magna]
7	KZS20885.1 JAN57391.1 JAN64473.1	NA	only in C	Uncharacterized protein APZ42_012329 [Daphnia magna] putative Lethal (1) G0193 [Daphnia magna] Lethal (1) G0193 [Daphnia magna]
8	KZS01522.1 JAN67828.1 JAN44234.1 JAN44235.1 KZS03198.1 KZS00546.1 JAN65939.1 (4 more)	0.02027	0.37	Vitellogenin fused with superoxide dismutase, partial [Daphnia magna] hypothetical protein [Daphnia magna] Copper-zinc cu-zn superoxide dismutase-like protein, partial [Daphnia magna] putative Copper-zinc cu-zn superoxide dismutase, partial [Daphnia magna] Vitellogenin fused with superoxide dismutase [Daphnia magna] Vitellogenin fused with superoxide dismutase, partial [Daphnia magna] hypothetical protein [Daphnia magna]
9	KZS03199.1 KZS00545.1 JAN61085.1 JAN53991.1 JAN44236.1 JAN44237.1 KZS01586.1 (20 more)	0.03012	0.61	Vitellogenin fused with superoxide dismutase [Daphnia magna] Vitellogenin fused with superoxide dismutase, partial [Daphnia magna] Copper-zinc cu-zn superoxide dismutase [Daphnia magna] hypothetical protein, partial [Daphnia magna] putative Copper-zinc cu-zn superoxide dismutase, partial [Daphnia magna] putative Copper-zinc cu-zn superoxide dismutase, partial [Daphnia magna] Vitellogenin fused with superoxide dismutase, partial [Daphnia magna]
10	KZS05119.1	0.03475	0.48	Acetyl-CoA acetyltransferase, cytosolic [Daphnia magna]

**Table 2:** Proteins with changed expressions in the N clone individuals which had been treated with CP, compared to control individuals, with q-value < 0.1 (ratio = control/CP treated animals).

no	protein	qvalue	ratio	description
1	JAN87821.1	NA	only in C	5-oxoprolinase [Daphnia magna]
2	JAN68607.1 JAN75681.1	NA	only in C	hypothetical protein [Daphnia magna] Trafficking protein particle complex subunit [Daphnia magna]
3	KZS17483.1	NA	only in C	Cadherin-23 [Daphnia magna]
4	JAN85428.1 JAN56086.1 JAN56087.1 JAN58286.1 JAN31693.1 JAN31694.1	NA	only in C	Transforming protein RhoA [Daphnia magna]
5	JAN88856.1 KZS09065.1	NA	only in C	Mannose-6-phosphate isomerase [Daphnia magna]
6	JAN66521.1 JAN74852.1 KZS17694.1	NA	only in CP	Cyclin-dependent kinase [Daphnia magna] Eukaryotic translation initiation factor 3 subunit F [Daphnia magna] Cdk1/cdc2-like protein [Daphnia magna]
7	JAN88015.1 KZS18876.1	NA	only in C	Integrin alpha-PS4 [Daphnia magna]
8	JAN48978.1 JAN51075.1 JAN36045.1 JAN38730.1 JAN36046.1 JAN45672.1 KZS12965.1 (2 more)	NA	only in CP	Vesicle-fusing ATPase [Daphnia magna]
9	KZS04920.1 JAN33178.1 JAN67728.1 JAN55609.1 JAN50414.1 JAN86991.1	NA	only in CP	Ribosome biogenesis protein WDR12 [Daphnia magna]
10	KZS02943.1 JAN56973.1 JAN53933.1 JAN49344.1 JAN47436.1 JAN30488.1 JAN45937.1 (2 more)	0.00240	1.52	Neprilysin (neutral endopeptidase) [Daphnia magna]
11	KZS07135.1 JAN87858.1	0.01443	0.76	V-type proton ATPase catalytic subunit A [Daphnia magna]
12	KZS13935.1 JAN57187.1 JAN51070.1 JAN42923.1 JAN30737.1 JAN70324.1	0.01603	1.38	Glutamate dehydrogenase, mitochondrial [Daphnia magna]

**Table 3:** Proteins with changed expressions in the S clone individuals which had been treated with CP, compared to control individuals, with q-value < 0.1 (ratio = control/CP treated animals).

no	protein	qvalue	ratio	description
1	JAN73747.1 JAN65254.1 JAN62759.1 KZS06757.1 JAN90719.1	NA	only in C	60S ribosomal protein L7, partial [Daphnia magna] 60S ribosomal protein L7 [Daphnia magna] 60S ribosomal protein L7, partial [Daphnia magna] Cytoplasmic FMR1-interacting protein 2 [Daphnia magna] Reelin [Daphnia magna]
2	KZS09908.1 JAN86372.1	NA	only in CP	Tetratricopeptide repeat protein 39C [Daphnia magna] Tetratricopeptide repeat protein 39A [Daphnia magna]
3	KZS08204.1 JAN49075.1 JAN58156.1 JAN44218.1 JAN70366.1 JAN49074.1 JAN47274.1 (1 more)	NA	only in C	Importin subunit alpha-1 [Daphnia magna] Importin subunit alpha-4 [Daphnia magna] Importin subunit alpha-4 [Daphnia magna] Importin subunit alpha-4 [Daphnia magna] Importin subunit alpha-4 [Daphnia magna] Importin subunit alpha-4 [Daphnia magna] Importin subunit alpha-4 [Daphnia magna]
4	JAN61523.1 JAN57486.1 JAN58519.1 JAN76102.1 KZS09622.1	NA	only in CP	Carboxypeptidase B2 [Daphnia magna]
5	JAN38586.1 JAN31331.1 KZS20920.1 JAN78036.1	NA	only in C	Oligomeric Golgi complex subunit 8 [Daphnia magna]
6	JAN62118.1 JAN34272.1 JAN59007.1 JAN29476.1	NA	only in CP	ADP/ATP translocase, partial [Daphnia magna]
7	JAN90467.1 JAN54081.1 JAN46034.1	NA	only in C	Tubulin beta-4B chain [Daphnia magna]
8	KZS04920.1 JAN33178.1 JAN67728.1 JAN55609.1 JAN50414.1 JAN86991.1	NA	only in CP	Ribosome biogenesis protein WDR12 [Daphnia magna]
9	JAN35737.1 JAN53431.1 KZS07904.1 JAN53432.1 JAN71433.1 JAN71531.1	NA	only in CP	Mannosyl-oligosaccharide glucosidase [Daphnia magna]
10	KZS01666.1 KZR95784.1 JAN79828.1 JAN67460.1 JAN61042.1 JAN45945.1 JAN57602.1 (32 more)	0.08474	1.08	Uncharacterized protein APZ42_001605, partial [Daphnia magna] Uncharacterized protein APZ42_010249, partial [Daphnia magna] hypothetical protein [Daphnia magna] hypothetical protein, partial [Daphnia magna] Vitellogenin-1 precursor [Daphnia magna] putative Vitellogenin-1 precursor [Daphnia magna] 39S ribosomal protein L22, mitochondrial, partial [Daphnia magna]

The proteins which appeared or increased significantly in abundance in CP-treated individuals from the D clone were identified as vitellogenin fused with superoxide dismutase and cytosolic acetyl-CoA acetyltransferase (Fig 4A, Table 1).

The proteins which appeared or increased significantly in abundance in CP-treaded individuals from the N clone were identified as cyclin-dependent kinase, vesicle-fusing ATPase, ribosome biogenesis protein WDR12 and V-type proton ATPase catalytic subunit A (Fig 4, Table 2).

The proteins which appeared or increased significantly in abundance in CP-treated individuals from the S clone were identified as tetratricopeptide repeat protein 39, carboxypeptidase B, ADP/ATP translocase, ribosome biogenesis protein WDR12 and mannosyl-oligosaccharide glucosidase (Fig 4A, Table 3).

In the CP-treated clone D individuals, proteins which were totally inhibited or decreased significantly in abundance were NADH dehydrogenase [ubiquinone] 1 alpha subcomplex subunit 2, vitellogenin-1 precursor-like protein, glutathione S-transferase theta-1 and ectonucleotide pyrophosphatase/phosphodiesterase (Fig 4B, Table 1).

In the CP-treated clone N individuals, proteins which were totally inhibited or decreased significantly in abundance were 5-oxoprolinase, trafficking protein particle complex subunit, cadherin-23, transforming protein RhoA, mannose-6-phosphate isomerase, integrin alpha-PS4, neutral endopeptidase (neprilysin) and mitochondrial glutamate dehydrogenase (Fig 4B, Table 2).

In the CP-treated clone S individuals, proteins which were totally inhibited or decreased significantly in abundance were cytoplasmic FMR1-interacting protein 2 (reelin), importin subunit alpha-4, oligomeric Golgi complex subunit, vitellogenin-1 precursor-like protein (Fig 4B, Table 3).

## Discussion

Cyclophosphamide is an alkylating agent used in anticancer therapy, which has also been found to be a contaminant of aquatic ecosystems. In this investigation, we studied the influence of CP on the life history parameters and the proteome of *D*. *magna*. Our approach of using low concentrations of contaminants to match their normal occurrence in the environment fits in with the latest research [16, 17]. This kind of study go beyond the mere assessment of the impacts of contaminants on mortality and reproduction and contribute to our understanding of the sub-lethal effects of anthropogenic chemicals at the cellular and molecular levels.

A previous study [23] found that after 21 days of exposure to CP, the Lowest Observed Effect Concentration (LOEC) in *Daphnia* was 100 mg L^-1^ and the No Observed Effect Concentration (NOEC) was 56 mg L^-1^. Warne and van Dam (2008) argued, however, that NOEC and LOEC data are flawed and should no longer be generated or used [24]. We agree with this assessment, and thus used CP at met in the environment low concentration of 10 ng L^-1^, but prolonged exposition. Pharmaceuticals, highly diluted in freshwater can be expected to act on organisms rather in modulatory way, with the tendency to strengthen the effect if supplemented over time.

In our study, following 5 days the exposure of *D*. *magna* to CP, we found no effect on growth rate (Fig 2) nor on size at first reproduction. Only one of the tested clones showed reactions to CP regarding age at first reproduction (clone S) and the number of neonates at first reproduction (clone N) (Fig 3). On the other hand, 5-day old *Daphnia* treated with CP differed in their proteome profiles compared to untreated, control animals (Fig 4); thus, an effect of CP was observed at the molecular level.

All tested clones showed different reactions to exposure to the alkylating agent. Given that low concentrations of CP as are typically found in the environment are not lethal, we took the opportunity to test for various strategies which *Daphnia* may use to cope with the negative effects of exposure to CP. *Daphnia* is well known for its phenotypic plasticity, particularly spectacular in the domain of sex. Males and females from the same clone have the same genotype and their sexual features are environmentally determined [23]. The different reactions to toxic substances by different clones can be a result of the species’ micro-evolutionary potential to develop resistance (in this case to CP). According to Messiaen et al. (2013), the initial tolerance shown by *D*. *magna* to sub-lethal cadmium exposure is the same among several naive populations living in different ponds, but the micro-evolutionary potential of these populations to develop resistance is very different [25]. It is likely that our clones had already been in contact with the alkylating agent. Given that they originated from populations which may have had different potentials to develop resistance, this may explain why they demonstrated such a wide range of reactions to CP.

The clones in our study reacted quite variably to the CP, and different proteins were affected in each. Nevertheless, we suggest that in general *Daphnia* attempt to counteract the cytotoxic activity of CP by increasing basic metabolism including the strategy of protein degradation using carboxypeptidase B. *Daphnia* also probably react to CP by regulating their cell cycles, e.g. through the activity of cyclin-dependent kinase (Tables 1–3).

In our clones, the absence or down-regulation of such proteins as glutathione S-transferase theta-1 and 5-oxoprolinase involved in glutathione synthesis, may stem from inhibition by the affected *Daphnia* of homeostasis in the redox state of their cells. In addition, *Daphnia* individuals treated with CP likely down-regulate the processes involved in cell adhesion, transport, and signaling, (e.g. trafficking protein particle complex, cadherin-23, importin, or ectonucleotide pyrophosphatase/phosphodiesterase) (Tables 1–3). In the cells of more complex organisms, overexpression of the aforementioned processes could lead to cancerogenesis.

It is known that CP induces double strand breaks in DNA that could possibly lead to apoptosis induction [26–28]. We did observe, in the D clone only, a nonsignificant decrease in the expression of mitochondrial apoptosis-inducing factor 1, clones (S1, S2, and S3 Files). The apoptosis-inducing factor 3 occurred at a decreased level in the D and S clones, but was not identified for N clone. On the other hand, the apoptosis inhibitor 5 decreased in the CP-treated D clone but increased in the CP-treated N and S *Daphnia*. This suggests that in all the clones, the process of apoptosis is restrained in the presence of low concentrations of CP; the N and S clones, however, may be less resistant to the chemical used.

D clone individuals showed no changes in their life history parameters when exposed to the alkylating agent tested. However, proteome analysis showed increase in the production of vitellogenin fused with superoxide dismutase. Chen et al. (2011) studied vitellogenesis in *Artemia parthenogenetica* in two reproductive modes, oviparity and ovoviviparity [29]. The vitellogenin of *Artemia* was found to possess six copies of the consensus cleavage site, R-X-X-R, and a superoxide dismutase (SOD)-like domain at the N-terminus. Different profiles of the oviparous and ovoviviparous pathways were shown to contain only the SOD-containing subunits. The authors postulated that these components play an important role in the formation of encysted diapaused embryos during long-term cell-cycle arrest. Previously, SOD-like domains for vitellogenesis proteins have been reported in only one crustacean, namely, *D*. *magna* [30, 31]. Under favorable environmental conditions, crustaceans of the *Daphnia* genus reproduce parthenogenetically. When conditions deteriorate, these organisms can switch to producing males and haploid sexual resting eggs [32]. We can speculate that animals of clone D interpret CP factor as indicating deteriorating environmental conditions and prepare themselves for diapause.

As described above, one way of dealing with toxic substances is to avoid it by producing resting stages able to survive any harsh conditions [33]. In our study, *Daphnia* of N clone appeared to take a different strategy. Individuals from this clone cultured in the presence of CP had many more offspring than those without the pharmaceutical (Fig 3C). Such a phenomenon has also been found to be produced by the presence of other stress factors, e.g. *D*. *magna* in the presence of fish kairomone allocate more energy for egg production [34]. In our study, the production of larger numbers of offspring may be an attempt by the *Daphnia* to compensate for higher offspring mortality resulting from a predicted toxic environment in the future. Moreover, based on their proteome profiles, the N clone animals exposed to CP underwent changes in their metabolic activity and cell organization, which may reflect an adaptation to the presence of a toxin in the environment.

The reaction of clone S represented another type of strategy taken by *Daphnia* exposed to CP. At the level of the individual, we observed a prolongation of the time necessary for *Daphnia* to reach maturity in the presence of the pharmaceutical, which can be explained by the configuration of the proteome profiles. Vitellogenin, the precursor of main yolk proteins, is crucial for embryonic development. Kato et al. (2004) found that vitellogenin 1 was the most abundant polypeptide in the parthenogenetic eggs of *D*. *magna* [30]. Our results showed decreased levels of the vitellogenin-1 precursor-like protein in the CP-treated *Daphnia*. The accumulation of vitellogenin, or vitellinis, in oocytes is one of the key events in the ovarian maturation process. In our experiment, *Daphnia* cultured with alkylating agent showed lower levels of the vitellogenin-1 precursor-like protein on the fifth day of incubation. As it takes longer to accumulate enough proteins to reach maturation, the *Daphnia* are thus older when releasing their first eggs.

Recently van Donk et al. (2015), showed multiple pathways by which pharmaceutical compounds may directly and indirectly affect species from different trophic levels [35]. Our research supports these conclusions. *Daphnia* is a model organism, a typical representative of the filter-feeding zooplankton which plays a major role in controlling algal biomass, and likely play an important role in limiting negative effects on ecosytems of eutrophication [36]. In addition, cladocerans including *Daphnia* constitute the primary food of planktivorous fish. Changes in any of their fitness parameters caused by the presence of pharmaceuticals in the environment could have a dramatic effect on these planktonic crustaceans, and thus on aquatic food webs. The structure and composition of the zooplankton community translate directly into the characteristics of the ichthyofauna which prey on them, and the health of these organisms is essential for fish production in lakes.

In conclusion, our study has demonstrated for the first time that even very low concentrations of CP in freshwater ecosystems can significantly impact aquatic organisms. This contaminant can influence both the life history parameters of as well as protein expression in *Daphnia*. Our different *Daphnia* clones employed different strategies to better survive in the contaminated environment. Nevertheless, our study provides a cautionary example of the effects of anthropogenic chemicals on aquatic ecosystems.

## Supporting information

S1 File Proteins with changed levels in *D*. *magna* D clone teated with cyclophosphamide. The differential analysis results of *D*. *magna* D clone protein profiles treated with cyclophosphamide, compared to the protein profiles of untreated individuals.
(XML)

S2 File Proteins with changed levels in *D*. *magna* N clone teated with cyclophosphamide. The differential analysis results of *D*. *magna* N clone protein profiles treated with cyclophosphamide, compared to the protein profiles of untreated individuals.
(XML)

S3 File Proteins with changed levels in *D*. *magna* S clone teated with cyclophosphamide. The differential analysis results of *D*. *magna* S clone protein profiles treated with cyclophosphamide, compared to the protein profiles of untreated individuals.
(XML)
