# Correction: How cyclophosphamide at environmentally relevant concentration influences *Daphnia magna* life history and its proteome

**DOI:** 10.1371/journal.pone.0197416

**Published:** 2018-05-10

**Authors:** Małgorzata Grzesiuk, Damian Mielecki, Tomasz Pilżys, Damian Garbicz, Michał Marcinkowski, Elżbieta Grzesiuk

In the Funding section, a grant number from the funder National Science Centre, NCN Poland, UMO is listed incorrectly. The correct grant number is: 2015/17/D/NZ8/00782. The correct Funding statement is: This work was supported by the National Science Centre, NCN Poland, UMO-2016/21/B/NZ8/01542 to EG and 2015/17/D/NZ8/00782. The funder had no role in study design, data collection and analysis, decision to publish, or preparation of the manuscript.
